# Exosomes as Intercellular Signaling Organelles Involved in Health and Disease: Basic Science and Clinical Applications

**DOI:** 10.3390/ijms14035338

**Published:** 2013-03-06

**Authors:** Chiara Corrado, Stefania Raimondo, Antonio Chiesi, Francesco Ciccia, Giacomo De Leo, Riccardo Alessandro

**Affiliations:** 1 Department of Biopathology and Biomedical and Forensic Biotechnologies, Section of Biology and Genetics, Università di Palermo, Palermo 90133, Italy; E-Mails: chiara.corrado@unipa.it (C.C.); stefania.raimondo@unipa.it (S.R.); giacomo.deleo@unipa.it (G.D.L.); 2 Exosomics Siena SpA, Siena 53100, Italy; E-Mail: antonio.chiesi@hansabiomed.eu; 3 Department of Internal and Specialistic Medicine, Section of Rheumatology, Università di Palermo, Palermo 90129, Italy; E-Mail: francesco.ciccia@unipa.it

**Keywords:** cancer microenvironment, exosomes, cell signaling, cancer markers

## Abstract

Cell to cell communication is essential for the coordination and proper organization of different cell types in multicellular systems. Cells exchange information through a multitude of mechanisms such as secreted growth factors and chemokines, small molecules (peptides, ions, bioactive lipids and nucleotides), cell-cell contact and the secretion of extracellular matrix components. Over the last few years, however, a considerable amount of experimental evidence has demonstrated the occurrence of a sophisticated method of cell communication based on the release of specialized membranous nano-sized vesicles termed exosomes. Exosome biogenesis involves the endosomal compartment, the multivesicular bodies (MVB), which contain internal vesicles packed with an extraordinary set of molecules including enzymes, cytokines, nucleic acids and different bioactive compounds. In response to stimuli, MVB fuse with the plasma membrane and vesicles are released in the extracellular space where they can interact with neighboring cells and directly induce a signaling pathway or affect the cellular phenotype through the transfer of new receptors or even genetic material. This review will focus on exosomes as intercellular signaling organelles involved in a number of physiological as well as pathological processes and their potential use in clinical diagnostics and therapeutics.

## 1. Introduction

The physiological cellular behavior within a normal tissue includes a regulated proliferation, adhesion, programmed cell death and ability to differentiate. It has become evident that a tight signal integration between growth factors and their receptors, cell-cell adhesion molecules, cell-matrix receptors, and intracellular signaling proteins, is required to coordinate all of these processes. Activation of one or more of these cellular components through growth factors and/or direct contact with molecules of the extracellular matrix (ECM) regulates intracellular signaling events resulting in cell behavior changes. In normal cells, these signals are purposefully switched on and off depending on the presence or absence of appropriate stimuli. As a consequence, the cell can dynamically modify its phenotype and then revert to its normal behavior when the stimulus ceases.

Schematically, signaling pathways start with receptor proteins commonly found at the cell surface, where they bind to extracellular molecules that cannot pass through the plasma membrane. Receptors can bind to signaling proteins such as growth factors and peptide hormones, small molecules such as nutrients, ECM proteins or other receptors located on adjacent cells. As a result of this interaction, a signal is transferred across the plasma membrane that may result in the stimulation of an activity that is integral to the intracellular part of the receptor. One of the most common activities is the phosphorylation of substrates by serine/threonine or tyrosine kinases. Other receptors may function as ion channels, allowing ions to flow into or out of the cell to control cellular functions. Alternatively, the part of the receptor within the cell may interact with other signaling proteins and transfer the information to them by inducing changes in conformation and activity. Cell-surface receptors such as integrins interact with ECM components or receptors on other cells thus playing a key role in establishing tissue architecture but also in informing cells of the presence of other cells and of the matrix. Finally, receptors inside the cell bind to signaling molecules that can penetrate the plasma membrane, such as steroid hormones and nitric oxide. Inside the cell, receptors pass on the signal to a variety of other molecules through the assembly of multimolecular signaling complexes that result in changes in the localization or activity of enzymes. Some proteins in these complexes, referred to as “docking” proteins, may work only to recruit other signaling molecules. Generally, signaling pathways end in the nucleus with a change in the gene-expression of the cell thus leading to the modulation of cell phenotype.

A number of pathologies, including cancer, are considered diseases of deranged signaling [1], therefore a greater knowledge of the strategies through which cells communicate is required to increase our weapons against different pathological disorders.

Cells are known to deliver proteins and molecules between the intracellular organelles via membrane vesicles containing definite receptors to ensure traffic specificity; the results accumulated over the last ten years have demonstrated that a heterogeneous group of vesicles are also released from the cell surface and used as intercellular signalosomes in information exchange, even over a long distance [2]. These membranous vescicles, released by a variety of cells and generally termed extracellular vescicles (EVs), can be divided into three main classes: exosomes, microvescicles and apoptotic bodies. Among these, exosomes have recently received most of the attention and will be discussed in this review. While exosomes are vescicles of endocytic origin and homogeneous in shape and size (40–100 nm), apoptotic bodies, released by cells undergoing programmed cell death, and microvescicles, are derived directly from the plasma membrane and show variable size (50–500 nm for apoptotic bodies, 100–1000 nm for microvescicles) and shape. EVs have been originally also named on the basis of their tissue origin; for example, prostasomes are vesicles secreted by prostate epithelial cells and secreted into the seminal fluid, argosomes are exosome-like vesicles isolated from *Drosophila melanogaster* and oncosomes, vescicles released by cancer cells [3].

A precise and clear distinction between these vesicles is still lacking, and in April 2012 in Goteborg a special committee of the newborn International Society of Extracellular Vesicles (ISEV) has been appointed to shed light on the nomenclature and methods to be used to distinguish between all of these membrane particles. In this review we will focus on exosomes and their role in health and disease.

## 2. Origin, Molecular Composition and Delivery of Exosomes to Target Cells

During the first years of 1980, many groups reported *in vitro* data on the release of vesicles containing enzymatic activities from different cell lines. Electron microscopy observations together with pulse-chase experiments also showed that, in differentiating reticulocytes, non lysosomal multivesicular endosomes were able to fuse back with the plasma membrane and release their inner content extracellularly, including several circular membrane fragments [4–6]. In 1987 the term “exosome” was coined to describe these small vesicles [7]. Exosome research was considered a highly specific field of investigation with almost no interest in the cell biology community until a paper by Raposo and colleagues showed that Epstein-Barr virus-transformed B-lymphocytes stimulate T cell proliferation by secreting exosomes containing MHC II dimers conjugated to antigenic peptides [8]. This seminal paper and others from the same group represented a milestone in this field, indicating that exosomes may be considered cellular structures used for cell-cell communication and not simple containers for waste disposal.

Exosomes are released *in vitro* by several cell types including B and T cells [9], dendritic cells [10], mast cells [11], mesenchymal stem cells [12], epithelial cells [13,14], astrocytes [15], endothelial cells [16] and cancer cells of almost all histotypes [17–21]. *In vivo*, exosomes have been isolated and characterized by a variety of body fluids such as plasma, urine, saliva, cerebrospinal, amniotic and synovial fluids [22–26].

The first step in exosome biogenesis is the formation of intraluminal vesicles (ILV) following the inward budding of the limiting membranes of late endosomes. These organelles, also called multivesicular bodies, move up to the cell surface, fuse with the plasma membrane and release the ILV outside the cell. The exosomes are between 40 and 100 nm in diameter, float at 1.1–1.19 g/mL in sucrose density gradient, have a particular cup-shaped morphology and, as showed by biochemical and proteomic analyses, have membranes enriched in special lipids (cholesterol, sphingomyelin and ceramide) and a unique protein composition that characterizes them as discrete organelles [3,27]. Although exosomes from different sources have a common set of proteins representing molecules that regulate membrane cytoskeleton dynamics and membrane fusion events such as the Rab family of GTPases [28], Alix and ESCRT (an endosomal sorting complex required for transport) proteins [29], a specific molecular signature that varies depending upon the nature and conditions of the cell type of origin is often observed. For example, the tetraspanins (CD81, CD9) and chaperons (HSP60, HSP70 and HSP90) that are involved in peptide loading on MHCI and II are often found in immune cell-derived exosomes [30], perforin and granzyme on cytoxic T-lymphocytes [31], transmembrane protein A33 on intestinal epithelial cells [32] and subunits of glutamate receptors on neurons [33]. A scientific debate is still ongoing to establish if a finely regulated cellular mechanism has evolved to determine the specific loading of the exosomes in the different cell types or if the exosome content is imposed by the unpredictable engulfment process during ILV biogenesis. Shen *et al.* identified plasma membrane anchors (e.g., myristoylation or palmitoylation tag) or different sequence motifs and structures (e.g., acylation sites or phospholipid binding domain) that target the proteins to exosomes [34,35]. Another element is the higher order oligomerization of target proteins: tetraspanins, one of the exosome marker proteins, are commonly found in large complexes that contribute to exosomes protein sorting pathway [36,37]. ESCRTs proteins are another example of exosomes cargo that are found at the plasma membrane and are located in membrane bound highly oligomeric protein complexes; it has recently reported that ESCRT-binding motifs induce the budding of proteins such as ARRDC1 (arrestin domain containing protein-1) and syntenin by recruiting a catalytic activity of the ESCTR machinery [38,39]. Alix, a protein associated with the ESCRT molecular apparatus, is specifically required for sorting the transferrin receptor into exosomes [40]. It remains to better elucidate how the cell recognizes protein that contain these signals and how signal containing proteins and/or protein complexes are targeted to exosomes.

Exosomes are enriched for specific nucleid acids, in particular miRNAs and RNAs that generally exist in complex with proteins, Ming Yang and Gould reported that selective trafficking of miRNA to endosomes can be mediated through RNP complexes; these complexes are transported towards late endosomes via kinesin and dynein motors, resulting into selective budding of their cognate RNAs [34]. For example, bicoid RNA binds ESCRT II complex and this interaction may allow the localization of the RNA in the endosomal system [41]; other studies have demonstrated that a subset of plasma miRNAs are bound to Ago2, which is often found inside the exosomes [42].

Exosome secretion can be constitutive or inducible depending on the cell type. Savina and colleagues showed that the release of exosomes from erythroleukemia K562 cells was markedly enhanced by MON treatment, a Na^+^/H^+^ exchanger that induces changes in intracellular calcium (Ca^2+^) implying a requirement for Ca^2+^ in this process [43]. Levine’s group demonstrated that mouse embryo fibroblasts with a wild-type *p53* gene produced exosomes after DNA damage but isogenic MEFs with no *p53* genes (from knockout mice) failed to produce exosomes after the same genotoxic stress. A p53-regulated gene product, TSAP6, was shown to be involved in exosome production thus alerting adjacent cells and the immune system of these events [44]. Once released in the extracellular space, exosomes interact with target cells inducing, according to the delivered molecules, a modulation of the phenotype toward a differentiated or activated state.

How exosomes may interact with a target cell is not yet fully known and several mechanisms have been hypothesized [2,32,45]. For example, exosomes can fuse with the target cell resulting in the non-selective transfer of proteins and RNA from exosome to the recipient cell. Parolini showed that exosome uptake from melanoma cells increases at a low pH and that this mechanism is dependent on the presence of sphingomyelin/ganglioside GM3. Moreover, they showed that fusion efficiency is higher with exosomes released from metastatic cells compared to those derived from primary tumors or normal cells [46]. Montecalvo and colleagues showed that dendritic cells (DCs) communicate with neighboring DCs through exosome-shuttle miRNAs, this cell-cell interaction was mediated via a “2-step event” consisting of exosome hemifusion with the cell membrane followed by the complete fusion of the exosomes with the limiting membrane of the phagosome; interestingly, they also observed that exosome content could vary according to the maturation of the parental DCs thus reinforcing the notion of an accurate selectivity in molecules sorted in exosomal cargos [47]. Exosomal membrane proteins can interact with the target cell in a juxtacrine fashion, acting as a ligand for cell surface receptors. Segura and colleagues studied the interaction between exosomes released by bone marrow-derived dendritic cells and CD8^+^ dendritic recipients cells showing that exosomes interact with DCs through a specific saturable receptor. Furthermore, they showed that exosomes secreted by mature DCs, bearing both functional MHC class II-peptide complexes and high amounts of ICAM-1 molecules, are captured and presented *in vivo* to non migrating murine CD8^+^ DCs through specific interactions with LFA-1, the naturally occurring counter-receptor of ICAM-1[48]. It is well established that rafts play an important role in signal transduction providing a well organized micro-environment, where the phosphorylation state of the resident proteins can be modified by local kinases and phosphatases, resulting in downstream signaling. Calzolari and colleagues demonstrated for the first time that activation of transferrin receptor 2 (TfR2) induces the activation of ERK1/2 and p38 MAPK pathways, supporting the hypothesis that TfR2 may function as a signaling receptor. In particular they showed that TfR2 is a new raft component sorted in exosomes and the localization of TfR2 in lipid rafts is essential for its signaling. Through the exosomal pathway TfR2 could act as an intercellular messenger, carrying a message about cell iron status [49]. Another example of receptor-ligand interaction mediated by exosomes on target cells is reported by Clayton and colleagues: using a panel of tumor cell lines, they showed that tumor exosomes may suppress a key tumor cell recognition pathway involving NKG2D, a receptor found in NK, NKT, CD8^+^ and gammadelta T cells. In particular they demonstrated that tumor exosomes carry NKG2D ligand, such as MICA and MICB, triggering a selective down regulation of cell surface NKG2D. These effects mediate suppression of lymphocyte functions, even in the presence of inflammatory cytokines such as IL-15, and consequently may mediate immune evasion in cancer [18].

In another study, the interaction between exosomes and the cell surface of target cells was mediated by an extracellular matrix component, fibronectin; B-lymphocytes also release exosomes that show high levels of integrin receptors on their membranes. The addition of these exosomes to TNF-alpha-activated fibroblasts triggered integrin-dependent changes in cytosolic calcium, measured by single cell imaging thus demonstrating not only that these receptors are fully functional but also that they may represent a novel mode of delivering adhesion signals over long distances[50].

Finally, a recent paper by Feng and colleagues showed that in phagocytic cells, exosomes can be internalized efficiently via phagocytosis [45]. Their experimental data demonstrated that exosomes adhered easily to the cell surface of non phagocytic cells but could not be internalized, as they could be removed by trypsinization or with extensive acid washing. In contrast, the same treatment did not remove exosomes from phagocytes, as they were already inside the cell [45].

## 3. Exosomes in Cell Physiology

Exosomes are now considered as an integral part of the intercellular microenvironment and may act as regulators of cell-to-cell communication. They may act as immune modulators with immunosuppressive or immune-activating effects by delivering proteins or nucleic acid to recipient cells. Exosomes, once released, may locally affect the behavior of target cells or enter in a biological fluid thus reaching distant sites. As our knowledge on exosome content and their modality of action increases, a higher number of physiological processes will be demonstrated to be regulated by these nanovesicles (Table 1).

### 3.1. Exosomes as Immune-Modulators

Since 1996, we have known that immune system cells release exosomes [8]. At first, immunologists supposed that exosomes might be extracellular organelles important to intercellular communication, with a potential role in immune-modulation. Currently, it is well known that exosomes contain several molecules used to vehicle messages between immune cells or between immune and target cells, and thus have an immunosuppressive or immune-activating effects on different steps of the immune response. Activated mouse DCs directly increase B cell effector functions through the release of exosomes, as evidenced by Wan and colleagues [69]. On the other hand, CD40 and IL-4 induce the release of exosomes in primary B cells, suggesting that B cells require proper activation for exosome production and that this process is strictly regulated by CD4^+^ T cells-derived stimuli [70].

The activation of T helper (Th) cells and the subseqent initiation of adaptative immunity are regulated by different types of antigen presenting cells (APC): in the absence of either self or pathogen-derived inflammatory products, DCs survey the antigenic environment and interact with T cells resulting in either tolerance or the regulatory cell’s induction. When infection occurs, antigen uptake by DCs increases and consequently the percentage of relevant MHC complexes, thus resulting in Th cell activation. In relation to MHC complexes, the activation of B cells by T cells (BCR/TLR or CD40/MHC II ligation) increases exosome production and releases MHC II complexes on B cell exosomes; the recycled MHC II complexes on exosomes directly stimulate CD4^+^ T cells, suggesting that B cell derived exosomes have a role as modulators of the immune response or as maintainers of antigen specific memory T cells [51]

Mature dendritic cells also secrete exosomes showing functional peptide-bearing MHC class I and II molecules on their membranesthat can directly bind to T-cell receptors and activate CD4^+^ or CD8^+^ T cells inducing an adaptive immune response [51,71]. As anticipated in the previous section, Segura and colleagues demonstrated that CD8^+^ DCs use LFA-1 to present exosomes in the lymph nodes confirming that exosomes might represent a vehicle for antigen transfer between DCs *in vivo*[48]. Subsequently, Nolte and collaborators demonstrated that the transfer of MHC class II-peptide complexes also occurs, *in vivo*, from DCs to CD4^+^ T cells, that DC derived exosomes are determinant for T-cell recruitment and that the T cell-DC interaction is mediated by high affinity LFA-1/ICAM-1 binding [52].

Exosomes secreted by immature or suppressive DCs reduce adaptive immune activation by inducing T cell apoptosis and thus promoting a tolerogenic immune response as seen in murine models of transplantation and autoimmune diseases. Suppressive exosomes may also influence the balance between pro-inflammatory and anti-inflammatory effector T cells inducing T helper Th17/Th1 cells to differentiate into Th2 and Foxp3^+^ regulatory T cells [72]. For this reason, exosomes could potentially be used to avoid graft rejection or as a treatment of autoimmune diseases. For example, in a mouse model of rheumatoid arthritis, the administration of exosomes derived from different DCs, genetically modified to express IL-4 or Fas-L reduced the clinical manifestation of the disease [73,74]; a study of Cai and colleagues demonstrated that immunosuppressive exosomes from TGF-β1 gene-modified DCs attenuated Th17-mediated inflammatory autoimmune disease by inducing regulatory T cells [75]. Recently, a potential role of exosomes as immune adjuvants has been demonstrated in transplants. For example, Peche and colleagues showed that the injection of immature DC-derived exosomes in rats induces tolerance and a prolonged survival time after a cardiac transplantation [76]; similar results were obtained by Yang and collaborators after an intestinal transplantation in rats [77]. A more complex approach was used by Li and colleagues which demonstrated that the combined treatment of exosomes derived from immature DCs and rapamycin can prolong cardiac allograft survival and induce specific allograft tolerance in mice [78]. Moreover, Montecalvo and colleagues showed that, after transplantation, graft-infiltrating leukocytes release exosomes but leukocytes also need DC derived-exosomes to stimulate donor reactive T cells in secondary lymphoid organs [79]. In conclusion, exosome-mediated cell-cell communication may constitute a potential mechanism by which DCs in lymphoid organs transfer alloantigens to effector cells and amplify the proliferation of donor-reactive T cell clones following transplantation.

### 3.2. Exosomes in Biologic Fluids

Exosomes are found in body fluids including plasma, urine, saliva, synovial fluid, breast milk, bronchoalveolar lavage fluid and epididymal fluid [26,80–87], supporting their *in vivo* role in physiological processes. Most of the studies have focused on the immunomodulatory abilities of these exosomes; the epithelial cells of the prostate gland release microvesicles in the seminal fluid, called prostasomes, which contain immunologically relevant proteins able to immunomodulate the microenvironment within the female reproductive tract. In particular, the authors showed their presence on prostasomes of CD48, a ligand for the NK-activating receptor CD244, supporting the idea that CD48–CD244 interaction might downregulate CD244 on NK cells, thus reducing their function. The immunomodulation of NK cell activity, mediated by prostasomes, is a new mechanism to increase sperm life-span, thus preventing immune-mediated sperm destruction and prolonging their survival rate [88]. During mammalian pregnancy, the maternal-fetal tolerance involves a number of immunosuppressive factors produced by the placenta; placental exosomes are involved in maternal immune surveillance and the recognition of the paternal antigens through the modulation of T-cell activity. Taylor and colleagues showed that exosomes isolated from the serum of pregnant women, who subsequently delivered at term, contained high levels of biologically active components, including Fas ligand (FasL) and HLA-DR. Fas signaling is a major mechanism for the induction of the peripheral clonal deletion of lymphoid cells. The authors correlated the release of exosomes expressing high levels of FasL with the pregnancies delivering at term, thus suggesting that Fas signaling might be one mechanism by which the placenta promotes the state of immune privilege essential for the birth [89]. Other authors also showed that Fas ligand is displayed on exosomes derived from trophoblasts inducing immune cell apoptosis thereby confirming the involvement of Fas ligand in the promotion of a state of immune privilege for the fetus, mediated by the downregulation of T cell activation [55,56]. Furthermore, cultured placental explants release exosomes containing NKG2D ligands able to selectively down-regulate the NKG2D receptor in CD8^+^ and gammadelta T cells, leading to the reduction of their *in vitro* cytotoxicity [57]. Interestingly, human and bovine milk exosomes contain bioactive molecules (protein, mRNA and miRNA) that can be transferred to immune cells to potentially modulate immune cell function and influence the immune system of the infant, promoting a state of immune privilege of the fetus [58,59,74,90,91]. Blood-borne exosomes with immunosuppressive or tolerogenic effects have been observed in different animal models. Kim and colleagues demonstrated that exosomes isolated from murine plasma were able to suppress the inflammatory response; the antiinflammatory effect was mediated by MHC class II-plasma exosomes and was also dependent upon the presence of FasL in the plasma exosomes [92]. On the other hand, plasma-derived exosomes isolated from antigen-immunized mice were shown to suppress Th1-type hypersensitivity as well as Th2-type allergic responses [53,54,93]. In line with these observations, more recently Prado and collaborators demonstrated that exosome-like vesicles, isolated from bronchoalveolar lavage fluid of BALF mice, can induce tolerance and protection against allergic sensitization thus indicating exosomes as an alternative to the conventional therapy for allergic diseases [94].

A number of data have been accumulated also for exosomes released into the urine from cells belonging to the entire renal tubule. Street and collaborators showed that urine exosomes may modulate nephron function; in particular they demostrated that murine cortical collecting duct cell-derived exosomes can transfer functional aquaporin to cells that did not previously express the protein thus resulting in a significant increase in water flow [95]. Proteomic studies of urine exosomes have suggested the processes in which exosomes may be involved; Wang and colleagues identified, through multidimensional protein identification technology (MudPIT), proteins that are known to play important roles in kidney function, such as proteins involved in water, drug, sodium, chloride, proton and glucose transport as well as some potential disease biomarkers for kidney diseases [96].

### 3.3. Exosomes and Genetic Materials: RNA Delivery and the Role of miRNA

The intercellular exchange of endogenous genetic material may occur in eukaryotic cells through several modalities including nanotubes, apoptotic bodies and nucleic acid- binding proteins [97]. In the last few years exosomes have been reported as containing not only proteins but also nucleic acids such as DNA, RNA, miRNA and long non coding RNA [61,98,99]. RNA-containing exosomes may represent an alternative pathway of cellular communication with significant implications in the modulation of cell phenotypes. The first experimental indication of the presence of mRNA and miRNA in exosomes from mouse and human mast cell lines was reported by Valadi and co-workers [61]; they showed that exosome mRNAs were functional, could be transferred in target cells and translated into proteins. Moreover, the gene profile analysis displayed differences in the quality of mRNA transcripts isolated from exosomes *versus* donor cells thus suggesting that a regulated mechanism of the intracellular sorting of exosomal mRNAs occurred in donor cells. Examples were let-7, miR-1, miR-15, miR-16, miR-181 and miR-375, which play a role in angiogenesis, hematopoiesis, exocytosis and tumorigenesis [61]. The authors subsequently showed, by affymetrix microarray analysis, which the exosomal mRNA content not only differs between exosomes and donor cells, but also between exosomes released from cells grown under different conditions such as serum deprivation or oxidative stress instead of normal conditions. Interestingly, these differences in mRNA content had a functional role since exosomes released by cells exposed to oxidative stress provided, in recipient cells, resistance to oxidative stress and cell death [100]. Other strong evidence for a selective sorting of miRNAs into exosomes comes from a recent study by Mittelbrunn *et al.* which demonstrated the existence of an antigen-driven unidirectional transfer of miRNAs from T cells to APCs that was mediated by the delivery of CD63^+^ exosomes [101]. Considering that specific miRNA populations are selectively sorted into exosomes, the exchange of genetic material may occur in the microenvironment close to the producing cells, but potentially also at a distance, through the systemic circulation in a endocrine-like way or through other body fluid types. For example, exosomes derived from human plasma can deliver RNAs to blood mononuclear cells such as monocytes and lymphocytes; Wahlgren and colleagues successfully introduced exogenous siRNAs into various kinds of human exosomes to deliver siRNA to human mononuclear blood cells. They demonstrated that plasma exosomes can deliver exogenous short interfering RNA (siRNA) to monocytes and lymphocytes causing the gene silencing of mitogen activated protein kinase 1 [25]. In relation to breast milk, human breast milk contains immune-related miRNAs capable to support the development of an infant’s immune system, as previously discussed. An analysis of miRNAs microarray from breast milk exosomes revealed the presence of T-cell-regulating miRNAs, in accordance with the role of human breast milk exosomes as immune modulators [84], miRNAs involved in B-cell differentiation, such as miR-181 and miR-155 [102–104] and the let-7-family of miRNAs, are all key miRNA regulators in development [60,105].

## 4. Exosomes in Pathology: Exosomes and Cancer

The evolution of a clinically significant invasive carcinoma requires an active collaboration of different actors found at the tumor-host interface: malignant epithelial cells, extracellular matrix, cancer-associated fibroblasts, inflammatory immune cells and normal mesenchymal cells [106].

**Figure 1:**
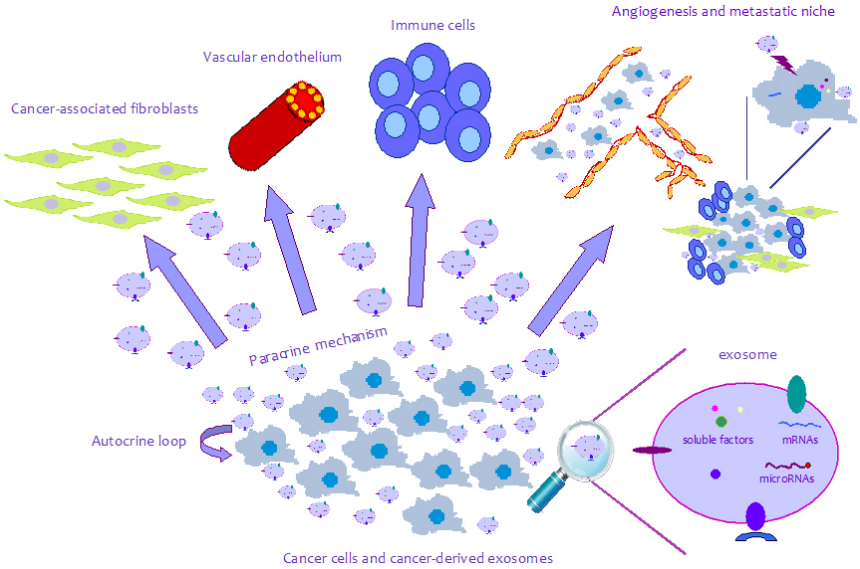
Schematic representation of exosome-mediated crosstalk. Tumor and normal stroma cells communicate through exosomes to establish a favourable tumor microenvironment and promote cancer growth, invasion and metastasis. Exosomes contain soluble factors, mRNA and miRNAs that may affect the phenotypes of different cytotypes such as fibroblasts, vascular endothelial cells and immune cells.

A variety of cytokines, growth factors, adhesion molecules and extracellular matrix proteins are secreted by both tumor and non-tumor cells, mediating cell-to-cell communication within the tumor microenvironment and providing a suitable niche for cancer cell growth and survival. A wealth of information has been obtained on the role these molecules and cell types play in promoting cancer invasion and metastasis. Recently, exosomes have been considered as new vehicles of these molecules into the tumor microenvironment and, for this reason, data is beginning to accumulate on their role as new actors in the crosstalk between cancer and normal cells in the tumor microenvironment (Table 1). Exosomes may function in an autocrine or paracrine manner to promote tumor-induced immune suppression, angiogenesis or premetastatic niche formation [19,107] (Figure 1).

Luga and collaborators have recently shown that cancer associated fibroblasts (CAFs) secrete exosomes that are able to promote, in an autocrine fashion, breast cancer cell protrusive activity and motility via Wnt-planar cell polarity (PCP) signaling. In particular, their results reveal a complex intercellular communication pathway, whereby CAF-secreted exosomes are internalized, modified and loaded with Wnt11 into breast cancer cells; exosomes are then released to activate an autocrine PCP signaling in breast cancer cells, thus promoting cancer cell migration and the acquisition of invasive behavior [62]. Bone marrow derived human mesenchymal stromal/stem cells (MSC), adjacent to primary tumor cells, have been shown to affect cancer progression by providing a favourable microenvironment and therefore promoting tumor growth, invasion and also metastasis through a process called premetastatic niche formation [108–111]. Recently, in fact, Zhu and colleagues have demonstrated that MSC release exosomes able to strongly trigger VEGF- and CXCR4-mediated pathways in tumor cells through the activation of Erk 1/2 and p38 MAPK kinases, thus resulting in an enhanced angiogenesis and tumor growth *in vivo*[112]. On the other hand, exosomes released by cancer cells could affect tumor growth, invasion and metastatic niche formation, angiogenesis and resistance to chemotherapy. Peinado and colleagues have recently shown that cancer-derived exosomes modulate the crosstalk between malignant cells and the bone marrow microenvironment; they reported, for the first time, that metastatic melanoma cells release exosomes that are able to “educate” bone marrow progenitors thereby inducing their mobilization which supports tumor vasculogenesis, invasion and metastasis, through the activation of the MET receptor tyrosine kinase. They identified that the MET-activated signaling proteins are expressed in highly metastatic melanoma derived-exosomes and that the transfer of the exosomal receptor tyrosine kinase MET from tumor derived-exosomes to bone marrow progenitor cells promotes the metastic process *in vivo*. These results suggest that bone marrow cells retain the educated phenotype after engraftment into a new host [19]. In lung cancer, Croce’s research group has reported that tumor derived-exosomes are enriched in particular miRNAs (miR-21, miR-27 and miR-29-a), that not only can be used as molecular markers of this cancer histotype but also act as paracrine agonists of the Toll like receptor (TLR) family in immune cells thus triggering a TLR-mediated prometastatic inflammatory response that induces tumor growth as well as the formation of secondary colonies at the metastatic site [63]. Several studies have revealed the role of cancer derived-exosomes in activating signal transduction pathways involved in cancer cell proliferation and survival. For example, Demory Beckler and colleagues detected KRAS in exosomes released by colon cancer cells and showed that the mutated KRAS can alter the signals induced by cells via exosomes, leading to a growth advantage in the recipient non-transformed wild-type KRAS-expressing cells [113]. Qu *et al.* have demonstrated that gastric cancer derived-exosomes promote, through an autocrine mechanism, the proliferation of tumor cells by PI3K/Akt and MAPK/ERK activation [114].

Although most of the studies on cancer exosomes have been done on solid tumors, little data has been produced for hematopoietic malignancies such as leukemia. Our group has reported that human Chronic Myelogenous Leukaemia (CML) cell lines such as LAMA84 and Imatinib-resistant LAMA84 cells as well as patients’ leukemic blasts, release exosomes that directly affect endothelial cells thus modulating the process of neovascularization. Specifically, the stimulation of vascular endothelial cells (HUVEC) with LAMA84-exosomes activate signal transduction pathways leading to the release of IL-8 and the induction of an angiogenic phenotype *in vitro* and *in vivo*[21,64]. Moreover, Mineo and colleagues have shown that exosomes, released from K562 CML cells, are internalized by HUVEC during tubular differentiation on Matrigel and move within and between nanotubular structures, connecting the remodeling endothelial cells; this exosome-induced angiogenic activity was mediated by Src kinase [65]. Exosomal miRNAs may have an important role in leukemia-endothelial cell communication; miR-92-a was detected in K562 derived- exosomes and has been demonstrated as having a role in enhancing endothelial cell migration and tube formation [115]. Recent data from Kurre’s group examined the role of exosomes released by Acute Myeloid Leukemia cells in modulating cell signaling in the bone marrow microenvironment. They showed that both primary AML blasts and AML cell lines released exosomes enriched in microRNA relevant to AML pathogenesis. In particular, they reported that the enrichment of miR-150 in AML derived-exosomes modified transcriptional responses and protein secretion in recipient cells; exosome transfer to Ba/F3 progenitor cells was, in fact, associated with a loss of CXCR4 surface expression and a consequent decrease in cell migration toward SDF-1α [66]. Hypoxia plays an important role in human tumor progression from *in situ* to metastatic cancers. Growing tumors are forced to survive in a poorly vascularized microenvironment characterized by hypoxia. To survive and grow in this hypoxic environment, tumor cells use adaptive mechanisms to promote proliferation, become resistant to apoptosis, induce angiogenesis, evade immune attack and migrate to less hypoxic areas; these mechanisms are mediated by the Hypoxia Inducible Factor (HIF) family of transcription factors [116,117]. Hypoxic tumor cells have been shown to release more exosomes to promote their own survival, angiogenesis and invasion; breast cancer cells exposed to a hypoxic environment enhance the production of exosomes in part due to HIF induction as shown using the HIF hydroxylase inhibitor dimethyloxalylglycine and siRNA interference. Interestingly enough, exosomes were also enriched in miR-210, a regulator of endothelial cell tubulogenesis and DNA repair pathways [118–121]. Other significant data were obtained through a quantitative proteomics study carried out on the A431 human epidermoid carcinoma. In this work, the authors reported that under hypoxia, cells secreted higher levels of proteins involved in angiogenesis, focal adhesion formation, extracellular matrix-receptor interaction and immune cell recruitment in comparison to cells undergoing normoxic and reoxygenation conditions. They found that more than 50% of these secreted proteins, predominantly classified as cytoplasmic and membrane proteins, were localized in exosomes [120].

Exosomes may indirectly contribute to tumor progression and metastasis development by interfering with the action of therapeutic agents, possibly through the transfer of mRNAs, miRNAs and/or proteins involved in drug resistance that drive the phenotypic changes of recipient cells. Exosomes may carry proteins involved in multidrug resistance, such us the P-glycoprotein or alternatively sequester the chemotherapeutic agent thereby decreasing the intracellular amount. Safei and coworkers demonstrated that cisplatinum-resistant ovarian cancer cells actively expelled anticancer drugs by enhancing the release of exosomes. Moreover, they reported that exosomes released by cisplatinum-resistant cells were enriched in cisplatinum and expressed higher levels of the transporters MRP2, ATP7A and ATP7B in comparison to sensitive cells [122]. Further studies showed that, in ovarian cancer cells, resistance to cisplatinum is also associated with an increased secretion of annexin A3, a member of the Ca^2+^ and phospholipid-binding annexin family, which prevents the uptake or accumulation of platinum in cells [123]. Electron microscopy observations have shown that annexin A3 detected in culture medium was localized in exosomes, revealing yet another exosome-mediated mechanism that affects a drug’s action [124]. Recently, Battke and colleagues have shown that exosomes can hamper the action of anticancer therapies by interfering with antibody-based drugs. The authors demonstrated that breast cancer cell lines over expressing HER2, release exosomes expressing a full-length HER2 molecule that is able to bind, both *in vitro* and *in vivo*, to the HER2 antibody, Trastuzumab, resulting in a reduced amount of antibodies available for the antibody-dependent cytotoxicity [125].

Data discussed in this section shows the key role of exosomes in modulating the tumor microenvironment: exosomes are not simply vehicles of molecules targeted into recipient cells, but also induce phenotypic changes in neighboring cells by activating specific cell signaling pathways, leading to cancer progression.

## 5. Exosomes in Pathology: Exosomes and Neurological, Cardiovascular and Rheumatologic Diseases

Exosomes have been proposed as being a way of intercellular communication in the normal physiology of the nervous system; several reports have demonstrated the release of exosomes by different cell types such as astrocytes [126] or microglial neurons [127]. Recently, Lachenal and collaborators demonstrated that exosomes released by differentiated neurons are regulated by synaptic glutamergic activity, and might thus be part of normal synaptic physiology [128]. Exosomes may also play a key role in neuronal communication during neurodegenerative diseases, such as Alzheimer’s, Parkinson’s or Prion diseases. Prion diseases are infectious neurodegenerative disorders linked to the accumulation of the abnormally folded scrapie prion protein (PrPsc) in the central nervous system [129]. In 2004, Fevrier and colleagues demonstrated that naturally occurring cellular prion proteins (PrPc) and PrPscs are released by cells in association with exosomes [130]; subsequently, exosomes from prion-infected neuronal cells have been demonstrated as being efficient initiators of prion propagation in uninfected recipient cells and, more importantly, producing prion disease when inoculated into mice [131]. Alzheimer’s and Parkinson’s disease are also characterized by the accumulation of misfolded proteins; recent studies have shown that the misfolded protein incorporation into exosomes protects them from degradation and also facilitates their delivery over long distances [132]. Parkinson’s disease is characterized by intracellular aggregates of α-synuclein, the Lewy bodies, in dopaminergic neurons. Alvarez-Ervit and colleagues showed that alpha-synuclein released from cells over expressing the protein is efficiently transferred to recipient normal cells through exosomes [133]. Moreover, Surgucheva showed that another member of the synuclein family, γ-synuclein, secreted from neuronal cells into exosomes can be transmitted to glial cells, thus promoting the aggregation of intracellular proteins [67]. Alzheimer’s disease (AD) is another neurodegenerative disorder, characterized by extracellular aggregates of beta amyloid peptides known as amyloid plaques [134]. In Alzheimer’s disease, exosomal proteins were found to accumulate in the plaques of AD patient brains, thus suggesting that exosome-associated-amyloid peptides may be involved in plaque formation with exosomes playing a significant role in the pathogenesis and the progression of AD [135]. Another characteristic of Alzheimer’s disease is the intraneuronal aggregation of abnormally modified microtubule-associated Tau proteins. Saman and colleagues have recently shown that tau proteins are mainly secreted through exosomes *in vitro* and *in vivo*[136].

Exosome-mediated cellular communication is also involved in the pathogenesis of cardiovascular disease. Kuwabara and colleagues reported that the serum levels of miR-1 and mir-133a, involved in the regulation of cardiac hyperplasia [137], are localized inside exosomes and are significantly increased in patients with acute myocardial infarction and angina pectoris [138]. Furthermore cardiovascular diseases have been associated with reduced vascular function and many studies have reported the presence of exosomes into the blood of patients with vascular disfunction. As shown by Pontes Azevedo, circulating platelet-derived exosomes from septic patients induced myocardial dysfunction in isolated heart and papillary muscle preparations in a nitric-oxide dependent mechanism [139]. Exosome-mediated cell-to-cell communication is also involved in atherosclerosis. Hergenreider and colleagues have recently reported that endothelial and smooth muscle cells (SMCs) communicate through exosomes which are enriched in miR-143/145; the expression of these miRNAs, in endothelial cells, is regulated by KLF2 (Krüppel-like factor 2), a key transcription factor able to mediate an atheroprotective endothelial phenotype generated by shear stress. They showed that these miRNAs are transferred into SMCs acting on miRNA targets thus preventing SMC de-differentiation [16].

Another emerging field is the role of exosomes in the disease progression of some autoimmune disorders such as Rheumatoid Arthritis (RA). RA is a chronic; systemic autoimmune disorder in which persistent synovial inflammation leads to joint destruction. Altered apoptosis sensitivity of fibroblast-like synoviocytes and T lymphocytes; leading to both synovial hyperplasia and chronic inflammation; have been extensively demonstrated in patients with rheumatoid arthritis (RA). Exosomes produced by synovial fibroblasts obtained from patients with RA contain a membrane form of TNF-α that; by inducing AKT and NF-κB; renders these cells resistant to apoptosis [68]. APO2L/TRAIL has been described as a TNF super family member capable of inducing apoptosis in tumor cells in a Fas-independent manner. Interestingly enough; the amount of bioactive APO2L/TRAIL associated with exosomes in synovial fibroblasts from RA patients appears to be low compared to that found in the synovial fibroblast of control patients. In this respect; Martinez-Lostao and co-workers have recently demonstrated that tethering APO2L/TRAIL to the liposome membrane (a surrogate of naturally occurring exosomes) increased its bioactivity; resulting in a more effective treatment of experimental arthritis compared with soluble; unconjugated APO2L/TRAIL; with substantially reduced synovial hyperplasia and inflammation in rabbit knee joints [140]. Over the past decade, it has become apparent that citrullinated proteins/peptides; and in particular the autoantibodies directed to them (anti-citrullinated protein antibodies (ACPA); are likely to be involved in the development of RA [141]. Synovial exosomes contain citrullinated proteins [26] and the expression of these proteins plays an important role in the induction and distribution of citrullinated proteins and presumably in the activation of those subsets of T cells that are specific to citrullinated proteins [142]. Altogether, these findings seem to suggest that a defective control of apoptosis by exosomes and their ability to promote T cell activation may play a role in the perpetuation of synovial inflammation in RA. In view of this evidence; answering some basic questions about the role of exosomes in RA becomes increasingly important. For example; little is known about the role of exosome in modulating the Th17 response; which seems to play a major role in the pathogenesis of RA. Thus, whether exosomes may be modulated by traditional disease modifying anti-rheumatic drugs and/or through biologic therapies remains to be determined as does the contribution of exosomes to immune responses in other rheumatic diseases such as Ankylosing Spondylitis and connective tissue diseases.

## 6. Exosomes in Clinical Diagnostic and Therapeutic Approaches

Exosomes, due to their cellular origin, their role in both physiologic and pathologic conditions and to recent technological developments that allow their selective capture, characterization and manipulation, are expected to significantly change many areas of clinical science. Clinical applications of exosomes have not yet hit the market place or routine medical practice, but pioneer applications both in diagnostics and in therapy are under development in several academic and industrial settings.

### 6.1. Diagnostics and Companion Diagnostics

The first attempts applying exosomes in clinical diagnostics were made several years ago. Due to their protein and nucleic acids contents, which closely reflects the nature and state of their parental cells, exosomes are considered veritable concentrations of information. All of the molecules found in exosomes are potentially useful for diagnostic/prognostic purposes (screening testing, confirmatory diagnostics, patient profiling and companion diagnostics, monitoring of treatments). Moreover, several viruses (HSV-1, EBV, HIV and many others) and infectious proteins (prions and prion-like proteins) have also been described as associated to exosomes. Recently published literature abounds with evidence in this regard, which is nicely summarized in recent reviews [143–145]. More data on exosomes associated proteins and nucleic cargos is also available on Exocarta (http://www.exocarta.org) [27] and on Vesiclepedia (http://www.microvesicles.org) [146].

The exosomal heterogeneous content, which is well protected by a lipid bilayer membrane that confers a high degree of stability, is enriched at its source thanks to specific sorting mechanisms, and includes some biomarkers that are otherwise scarce and under detected using traditional approaches [147]. Of course, due to their nanoscale dimension, not all exosomes, even if originating from a single cell, carry exactly the same amount and quality of proteins and nucleic acids [148]. The abundance of exosomes of different origins and different compositions in peripheral blood makes a further enrichment of the relevant biomarkers for a specific condition via a selective immune-capture of the exosomes of a specific origin, *i.e.*, coming from a specific tissue or expressing a specific biomarker [3], necessary when considering exosomes for diagnostic or companion diagnostic purposes. Several strategies for the efficient selective immune-capturing of exosome subpopulations are currently being verified (company data not shown). Targeting exosome associated biomarkers in different pathologic conditions does not only allow the discovery of novel biomarkers, but also gives unexpected new value to well-known disease biomarkers such as PSA (company data not shown). Much progress has been made in oncology where exosome-derived miRNAs have been associated with disease progression. For example, Rabinowits and colleagues showed the significant difference in total exosome and miRNA levels between lung cancer patients and controls, suggesting that circulating exosomal miRNAs might be useful as a screening test for lung adenocarcinoma [149]. Moreover, Taylor and colleagues showed that microRNA signatures of tumor-derived exosomes can be used as diagnostic biomarkers of ovarian cancer [150].

Recent proteomic studies have indicated that exosomes from cancer patients show specific protein patterns, thus indicating that the protein composition of exosomes might be useful for the early detection of various cancers. For example, a proteomic analysis of urinary exosomes allowed the identification of eight proteins that were potential bladder cancer biomarkers [151]. Liang and collaborators performed a high quality proteomic study of ovarian cancer-derived exosomes. The gene ontology (GO) analysis results revealed that exosomes may carry tissue specific proteins, which may provide some new biomarkers for the diagnosis of ovarian cancer [152]. Through the same approach, Chen and colleagues have recently identified differentially expressed proteins in urinary exosomes as novel candidate biomarkers for the discrimination of low-grade and high-grade bladder cancer [153]. Advances in technologies in exosome capture and their characterization will bring this important area to the clinic in the next few years.

### 6.2. Therapy and Regenerative Medicine

Interest in using exosomes in therapeutic approaches as well as evidence of their efficiency is an even more recent development. In these applications, exosomes are mostly regarded as a new delivery system for several therapeutic agents and for cell-free approaches in regenerative medicine. The use of natural autologous exosomes from MSC or synthetically created exosome mimetic nanoparticles is currently under investigation and has attracted a great deal of interest and expectations. Several functional studies available in recent literature have demonstrated that exosomes have a specific cell tropism, according to their characteristics, which can be used to target them to specific tissues and/or organs. This interaction causes the modulation of phenotype of target cells through a mechanism which is similar to a cellular signaling based response. Moreover, exosomes or exosome mimetic nanoparticles can be engineered and/or loaded with several molecules (drugs, small molecules, oligos, nanoparticles) and targeted to specific organs, and therefore can be used for the delivery of therapeutic agents in a targeted manner while enhancing the stability of the molecular cargo and protecting it from metabolic degradation.

In 2010, Alvarez-Erviti *et al.* first described the possibility of delivering siRNA to a mouse brain via a systemic injection of targeted exosomes, thus postulating a potential therapeutic approach to Alzheimer’s disease [154]. A nice protocol for the generation of targeted exosomes through the transfection of an expression vector, the loading of modified vesicles with BACE1 siRNA and their use to efficiently deliver siRNA *in vitro* and *in vivo* into the mouse brain was published by the same group in 2012 [155]. Ohno *et al.* showed that systemically injected exosomes targeted to EGFR-expressing breast cancer cells may deliver Let-7a antitumor miRNA, and thus represent a vehicle for conveying drugs to tumors [156]. Wahlgren *et al.* described that human plasma exosomes can be used as gene delivery vectors of siRNA to T cells and monocytes, suggesting their use in gene therapy provides target cells with heterologous siRNA [25]. The mentioned examples show that exosomes are currently considered good vehicles to shuttle oligonucleotides for therapeutic approaches, despite some technological, functional and safety features that are still to be addressed.

Other studies have also provided good evidence of the possibility of loading exosomes with conventional drugs. For example, in 2010 Sun *et al.* demonstrated that exosomes can be used as a vehicle to improve the solubility, stability and bioavailability of curcumine, an anti-inflammatory agent, and to enhance the delivery of the drug to activated monocytes, with an overall improved anti-inflammatory activity of the drug, in the absence of any side effects [157].

Also, in the field of regenerative medicine, due to the exosome’s afore-mentioned capabilities of altering the status of recipient cells and to the lack of evidence that cells employed to regenerate damaged solid organs give rise to any significant replacement of organ-specific cell populations, the concept of using a cell-free approach by injecting exosomes into the host instead of engrafting cells, is raising interest within the scientific community. The role of paracrine factors and mechanisms in mediating the positive results reported in several pre-clinical and clinical studies is currently central in the scientific debate. Nice reviews from Lai [158] Biancone [159] and Ratajczak [160] effectively discuss these issues and offer a future perspective in the field. The group of Lim in Singapore, working on acute myocardial infarction repair, has recently proposed a model consisting of MYC transformed human embryonic stem cell-derived MSC a scalable manufacturing process to obtain exosomes either as therapeutic agents or as delivery vehicles [161].

Even though all of the studies presented above do clearly indicate the feasibility of using exosomes for therapeutic approaches and pave the way for their clinical use, there are still several issues to be addressed and solved before such solutions can be widely proposed in clinical practice. Among these issues, a better understanding is needed of the organ/tissue specific tropism of different sub-populations of exosomes, the mechanisms of cell uptake, the efficiency of targeting strategies, the degree of integrity of the shuttled cargo, the degree of interference between the natural exosomes cargo and the artificially loaded one. Moreover, clinical translations of exosome based therapeutic approaches also require the development of scalable generations of exosomes or exosome mimetic particles for human use and/or cost effective methods of generating scalable patient-derived exosomes.

### 6.3. Immunotherapy and Vaccines

Already, over a decade ago, patents for using exosomes for vaccines were filed by several companies and several clinical trials using exosomes in cancer vaccine strategies were conducted; they were shown to be safe and potent inducers of an immune response against tumors. Since 1998, Zitvogel *et al.* have proposed the use of cell-free vaccines in tumors, showing that DC-derived exosomes promote a T cell-dependent antitumor immune response *in vivo*[71] and, in subsequent years, the same group supported their theory with further evidence and elucidated the mechanisms of CTL priming by exosomes *in vivo*[162,163]. In line with this, Cho *et al.* reported the use of exosomes engineered to express a specific tumor antigen to generate an immune response against tumors, showing that tumor-cell derived exosomes stimulate immune system and reduce tumor growth [164].

Similarly, exosome based vaccines have also begun to be considered and proposed in several vaccine orphan infectious diseases [165]. Martin-Jaular *et al.* have shown that exosomes from Plasmodium yoelii-infected reticulocytes protect mice from lethal infections, thereby opening new avenues for the modulation of anti-malaria responses [166], while Nanjundappa *et al.* have shown that a GP120-specific exosome-targeted T cell-based vaccine is capable of stimulating DC- and CD4^+^ T-independent CTL responses and may be useful in the induction of efficient CTL responses in AIDS patients with DC dysfunction and CD4^+^ T cell deficiency [167]. In their recent review Yin *et al.* analyze and discuss the possible use of immature DC-derived exosomes as a subcellular vaccine in autoimmunity [72]. Finally, in a recent original paper, Lattanzi *et al.* describe and propose a new method of incorporating protein antigens in exosomes, relying on the unique properties of a mutant of the HIV-1 Nef protein, a biologically inactive mutant they found by incorporating it into exosomes at high levels while also fusing at its *C*-terminus with foreign proteins [168].

Despite the nice and successful experimental evidence mentioned above (as well as other evidence unmentioned here), so far a vaccine approach in cancer has been limited by the fact that exosomes must be prepared locally using the treated patient’s dendritic cells, thus causing on one side variation in the yield of tumor antigen-loaded exosomes and therefore variation in the amount of treatment received, and on the other side a scalability problem because this personalized approach inhibits the wide diffusion of such kinds of treatments. Moreover, applications in the field of infectious disease are still immature, with only early reports and pilot studies. In the future, technological advancements in exosome manipulation/bioengineering or in the use of synthetic exosome mimetic nanoparticles, together with a better knowledge of exosome characterization and physio/pathology may solve those problems that currently limit the adoption of exosome based vaccine strategies to diseases and allow a wider diffusion of this approach.

## Figures and Tables

**Table 1:** Functions of tissue derived-exosomes in physiology and pathology.

Tissue/cell derived-exosomes	Target	Function	References
Activated B cells	CD4^+^ T cells	Modulation of immune response and maintaining antigen specific memory T cells	[51,52]
Mature DCs	CD8^+^ T cells	Vehicle of antigen transfer between different DCs	[47]
Plasma	T cells	Suppression of Th1-type hypersensitivity response; suppression of Th2-type allergic response	[53,54]
Plasma	Monocytes and lymphocytes	Delivering of specific exogenous siRNAs targeting MAPK pathway	[25]
Placenta	Fetus and child	Modulation of T cell activity; immune surveillance and recognition of paternal antigens	[55–57]
Breast milk	Infant	Modulation of infant’s immune cell function via miRNAs involved in T cell regulation and B cell differentiation	[58–60]
Mouse mast cells	Primary bone marrow-derived mouse mast cells	Regulated exchange of genetic material (mRNAs and miRNAs)	[61]
Cancer Associated Fibroblasts (CAFs)	Breast cancer cells	Promotion of cells’ protrusive activity and motility	[62]
Metastatic melanoma cells	Bone marrow progenitor cells	Support of tumor vasculogenesis, invasion and metastasis through MET	[19]
Lung cancer cells	Toll Like receptor (TLR) family in immune cells	Activation of prometastatic inflammatory response through specific miRNAs	[63]
Chronic myelogenous leukaemia cells	Human vascular endothelial cells	Induction of an angiogenic phenotype through the release of IL8 (21, 110) or activation of Src Kinase (111)	[21,64,65]
Acute myeloid leukaemia blasts and cells	Ba/F3 progenitor cells	Alteration of proliferative, angiogenic and migratory responses through specif miRNAs	[66]
Neuronal cells	Glial cells	Transmission of γ-sinuclein thus promoting the aggregation of intracellular protein	[67]
Endothelial cells	smooth muscle cells (SMCs)	Transfer of specific miRNAs thus preventing SMC de-differentiation	[16]
Synovial fibroblast from AR patients	CD4^+^ T cells	Induction of AKT and NF-κB pathways leading to apoptosis resistance	[68]
